# Progress and Prospects of Non-Canonical NF-κB Signaling Pathway in the Regulation of Liver Diseases

**DOI:** 10.3390/molecules27134275

**Published:** 2022-07-02

**Authors:** Li Tao, Xiaomeng Ren, Wenhui Zhai, Zheng Chen

**Affiliations:** 1 Emergency Department, 305 Hospital of People’s Liberation Army, Beijing 100017, China; taolistar@163.com (L.T.); pla305hos@126.com (W.Z.); 2 College of Pharmaceutical and Biology Engineering, Shenyang University of Chemical Technology, Shenyang 110142, China; 3 HIT Center for Life Sciences, School of Life Science and Technology, Harbin Institute of Technology, Harbin 150001, China

**Keywords:** non-canonical NF-κB signaling pathway, NF-κB-inducing kinase, liver diseases, NF-κB2, metabolism

## Abstract

Non-canonical nuclear factor kappa B (NF-κB) signaling pathway regulates many physiological and pathological processes, including liver homeostasis and diseases. Recent studies demonstrate that non-canonical NF-κB signaling pathway plays an essential role in hyperglycemia, non-alcoholic fatty liver disease, alcoholic liver disease, liver regeneration, liver injury, autoimmune liver disease, viral hepatitis, and hepatocellular carcinoma. Small-molecule inhibitors targeting to non-canonical NF-κB signaling pathway have been developed and shown promising results in the treatment of liver injuries. Here, the recent advances and future prospects in understanding the roles of the non-canonical NF-κB signaling pathways in the regulation of liver diseases are discussed.

## 1. Introduction

The liver is a key organ with multiple functions, including maintaining blood glucose and amino acid levels, synthesizing bile, storing key nutrients (e.g., glycogen and triglyceride), and detoxifying drugs and endotoxins. Maintaining normal liver function is important for maintaining whole-body homeostasis. Liver homeostasis is tightly controlled by nutrients, hormones, and multiple signaling pathways. Due to its key function as a filter for all of the blood in the body and defending the body against toxic threats, the liver is easy to damage. These threats, including over-nutrition, obesity, alcohol, viral infections, drugs/toxins, autoimmune disorders, genetic risks, and cancer, result in many liver diseases such as metabolic liver diseases, non-alcoholic fatty liver disease (NAFLD), non-alcoholic steatohepatitis (NASH), alcoholic fatty liver disease (AFLD), chronic liver injury, viral hepatitis, autoimmune hepatitis (AIH), liver cirrhosis and hepatocellular carcinoma (HCC). Multiple signaling pathways contribute to liver diseases.

Recent studies indicate that the non-canonical nuclear factor kappa B (NF-κB) signaling pathway is a key regulator of liver diseases. Great progress has been made to elucidate how abnormal activation in the non-canonical NF-κB signaling pathway drives liver diseases, from metabolic disorders to HCC [1,2,3,4,5]. Therefore, many researchers have turned to the non-canonical NF-κB signaling pathway as a potential therapeutic target for liver injury and inflammation [6,7]. This review summarizes the current understanding of the non-canonical NF-κB signaling pathway in the pathogenesis of liver diseases.

## 2. Non-Canonical NF-κB Signaling Pathway

**Figure 1:**
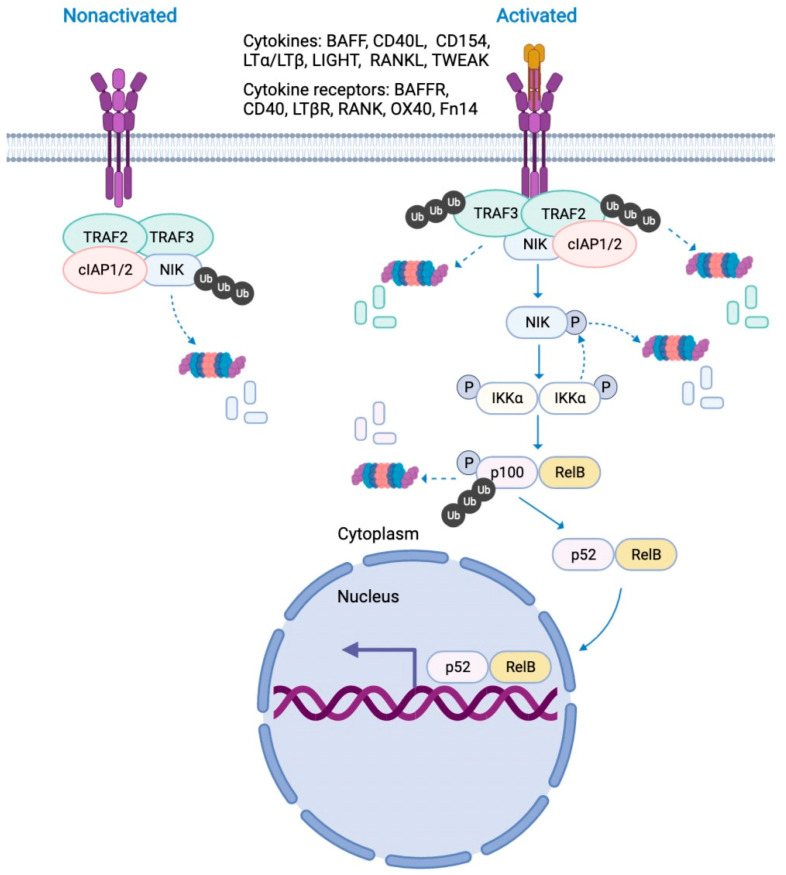
The non-canonical NF-κB signaling pathway. Under physiological conditions, the non-canonical NF-κB signaling pathway is inactivated because of the low levels of NIK protein, which is due to TRAF2-, TRAF3-, and cIAP1/2- mediated ubiquitination and degradation. Under stress or pathological conditions, elevated cytokines such as BAFF, CD40L, CD30L, LTα/β/LIGHT, OX40L, RANKL, and TWEAK bind to their receptors BAFFR, CD40, CD30, LTβR, OX40, RANK, and Fn14, respectively, which recruit TRAF2, TRAF3, and cIAP1/2, causing their ubiquitination and degradation. NIK protein levels are increased, and NIK phosphorylates IKKα and NF-κB2, leading to p100- to p52- processing. p52 and RELB form heterodimers and enter the nucleus to perform their function by activating gene transcription. BAFF: B-cell-activating factor; CD40L: CD40 ligand; CD30L: CD30 ligand; LTα/β: lymphotoxin alpha/beta; OX40L: OX40 ligand; RANKL: receptor activator of NF-κB ligand; TWEAK: tumor necrosis factor-like weak inducer of apoptosis; BAFFR: B-cell-activating factor receptor; LTβR: lymphotoxin beta receptor; RANK: receptor activator of NF-κB; Fn14: FGF-inducible 14; TRAF2: TNF-receptor associated factor 2; TRAF3: TNF-receptor associated factor 3;cIAP1/2: cellular inhibitor of apoptosis 1/2; Ub: ubiquitin; NIK: NF-κB-inducing kinase; IKKα: inhibitory kappa B kinase alpha.

The NF-κB transcription factors, including RELA (p65), c-REL, RELB, NF-κB1 (p50 and its precursor p105), and NF-κB2 (p52 and its precursor p100), are inactivated in the cytoplasm of quiescent cells [8]. Two different signaling pathways (canonical and non-canonical) can activate the transcription factor NF-κB in response to different stimuli [9,10]. The canonical NF-κB signaling pathway mediates the activation of NF-κB1 p50, RELA, and c-REL in response to stimuli from diverse immune receptors, which has been well studied [10]. The non-canonical NF-κB signaling pathway specifically activates NF-κB2 p52 and RELB. NF-κB-inducing kinase (NIK) is the key activator of the non-canonical NF-κB signaling pathway [9]. Under physiological conditions, the non-canonical NF-κB signaling pathway is inactivated because of the low levels of NIK protein, due to TNF-receptor associated factor 2/3 (TRAF2/3)-and cellular inhibitor of apoptosis 1/2 (cIAP1/2)- mediated ubiquitination and degradation (Figure 1) [11]. Under stress or pathological conditions, elevated cytokines such as B-cell-activating factor (BAFF), CD40 ligand (CD40L), CD30 ligand(CD30L), lymphotoxin alpha/beta (LTα/β), LIGHT, OX40 ligand (OX40L), receptor activator of NF-κB ligand (RANKL), and tumor necrosis factor-like weak inducer of apoptosis (TWEAK) bind to their receptors BAFF receptor (BAFFR), CD40, CD30, lymphotoxin beta receptor (LTβR), OX40, receptor activator of NF-κB(RANK), and FGF-inducible 14 (Fn14), respectively. In turn, these receptors recruit TRAF2, TRAF3, and cIAP1/2, causing their ubiquitination and degradation (Figure 1) [9]. Therefore, NIK protein levels are increased, and NIK phosphorylates inhibitory kappa B kinase alpha (IKKα) and NF-κB2, leading to p100- to p52- processing [12,13,14]. p52 and RELB form heterodimers and enter the nucleus to perform their function by activating gene transcription [9].

## 3. The Non-Canonical NF-κB Signaling Pathway Regulates Liver Diseases

Recent studies have demonstrated that key molecules in the non-canonical NF-κB signaling pathway regulate liver homeostasis and diseases, including metabolic liver diseases, NAFLD, NASH, AFLD, toxin-induced liver injury, liver regeneration, hepatic ischemia/reperfusion injury (HIRI), viral hepatitis, AIH, and HCC (Table 1).

### 3.1. Hepatic Glucose Disorder in Obesity

The liver controls blood glucose levels mainly through hepatic glucose production (HGP), including glycogenolysis and gluconeogenesis, which are regulated by insulin and glucagon in response to feeding and fasting. In obesity and type 2 diabetes, gluconeogenesis is abnormally elevated, thus contributing to hyperglycemia and glucose intolerance. In obesity, key molecules in NIK signaling pathway such as BAFF, CD40L, TRAF2, TRAF3, NIK, and p52 are abnormally elevated [1,2,15,18,20]. Although plasma cytokines such as BAFF and CD40L are significantly elevated in obesity [15,18], TRAF2 and TRAF3 are still highly expressed in the obese liver, which is likely due to the activation of their transcription [1,2]. The negative regulation of NIK by TRAF2 and TRAF3 is disrupted in the obese liver mainly because inflammation, oxidative stress, and steatosis (TNFα, H_2_O_2_, and palmitic acid) can activate NIK in hepatocytes [20]. The abnormally elevated key molecules in the NIK signaling pathway contribute to hyperglycemia and glucose intolerance in obesity. Deletion of either *BAFF* or *BAFFR* improves glucose tolerance in high-fat diet (HFD)-induced obesity [16,17]. Hepatic deletion of *TRAF2* protects against HFD-induced hyperglycemia by decreasing hepatic glucose production without affecting insulin sensitivity [1]. Hepatic deletion of *TRAF2* impairs the glucagon/p-CREB/G6pase/PEPCK signaling pathway, whereas overexpression of TRAF2 promotes this signaling pathway. Interestingly, hepatic deletion of *TRAF3* protects against HFD-induced glucose intolerance by increasing insulin sensitivity [2,3]. However, mice with hepatocyte-specific deletion of *NIK* show normal glucose homeostasis in either the normal chow diet or high-fat diet (HFD) conditions [21]. In contrast, deletion of *NIK* in the liver, including both hepatocytes and immune cells, protects against HFD-induced glucose intolerance [21], which indicates that hepatocyte NIK and immune cell NIK act together to promote hepatic glucose production in obesity. Myeloid cell-specific deletion of *TRAF3* also protects against HFD-induced glucose intolerance [19]. All these tissue-specific knockout mouse data demonstrate that the hepatocyte NIK signaling pathway and immune cell NIK signaling pathway work together to promote hepatic glucose production in obesity.

### 3.2. Non-Alcoholic Fatty Liver Disease

Aside from hyperglycemia and glucose intolerance in obesity, NAFLD is another metabolic liver disease, which ranges from non-alcoholic fatty liver (NAFL) to NASH. Worldwide, about a quarter of the population has a NAFLD, and about 25% of patients with NAFL develop NASH, characterized by hepatic steatosis, liver injury, chronic inflammation, and liver fibrosis, a key step in the pathogenesis of cirrhosis and HCC. In NAFLD, key molecules in the non-canonical NF-κB signaling pathway such as BAFF, CD40L, TRAF2, TRAF3, NIK, and p52 are abnormally elevated [1,2,15,18,20]. The abnormally elevated key molecules in the non-canonical NF-κB signaling pathway contribute to NAFLD. Deletion of *BAFF* or *CD40L* attenuates HFD-induced NAFLD by decreasing de novo lipogenesis and fatty acid uptake [16,22]. However, deletion of their receptors *BAFFR* or *CD40* promotes HFD-induced NAFLD [17,23]. Cytokines (BAFF and CD40L) and their receptors (BAFFR and CD40) show different roles in the pathogenesis of NAFLD, which may be due to different molecular mechanisms. The generation and phenotyping of hepatocyte-specific *BAFFR* or *CD40* knockout in mice is a good approach further clarifying the role of BAFFR or CD40 in NAFLD progression. Either hepatocyte or myeloid cell-specific deletion of *TRAF3* protects against HFD-induced NAFLD [2,3,19], which indicates that TRAF3 in either hepatocytes or myeloid cells are essential for the pathogenesis of NAFLD. Deleting *NIK* in the liver, including hepatocytes and immune cells, reduces HFD-induced liver steatosis by suppressing liver inflammation and lipogenic programs. However, *NIK* knockout in hepatocytes or immune cells alone does not alleviate HFD-induced hepatic steatosis [21]. Similarly, hepatocyte-specific deletion of *TRAF2* does not ameliorate HFD-induced NAFLD or inflammation. These results suggest that TRAF2/NIK in hepatocytes is not required for the pathogenesis of NAFLD, and immune cells play important roles in HFD-induced NAFLD. NIK signaling pathways in different cell types, such as hepatocytes, Kupffer cells, and other immune cells, may work together to promote HFD-induced NAFLD.

### 3.3. Alcoholic Fatty Liver Disease

In addition to NAFLD, alcohol consumption also induces steatosis, liver damage, and inflammation [62]. In alcoholic liver disease, inflammation is an essential driver in the initiation and progression of alcoholic steatosis. Although NIK is not essential for HFD-induced NAFLD [21], NIK promotes alcoholic steatosis via inhibition of fatty acid oxidation by suppressing hepatic PPARα. Under chronic alcohol administration conditions, hepatic steatosis is induced. NIK and p52 levels are elevated in the liver [5,24]. Hepatocyte-specific ablation of *NIK* ameliorates alcoholic steatosis in mice by maintaining fatty acid oxidation [24]. The detailed molecular mechanisms and whether other key molecules in this signaling pathway play important roles in AFLD need further study.

### 3.4. Toxin-Induced Liver Injury

Toxin-induced liver injury is a common liver disease. Toxins such as drugs, herbals, dietary supplements, lipopolysaccharide (LPS), and carbon tetrachloride (CCl_4_) damage hepatocytes by increasing cell membrane permeability, concentrations of highly reactive free radicals, and expression of proinflammatory cytokines including LTβand CD40L, ultimately leading to severe apoptosis and necrosis [28,63,64,65,66,67,68,69,70,71].This process is associated with the activation of the non-canonical NF-κB signaling pathway [5,6,26,28,70,71]. In the choline-deficient, ethionine-supplemented (CDE)-induced liver injury murine model, LTβ is highly expressed by liver progenitor cells (LPCs) [25]. In response to LTβ and LIGHT, LTβR activates the non-canonical NF-κB signaling pathway in hepatic stellate cells [25]. *LTβR* knockout in mice protects against CDE diet-induced liver fibrosis, inflammation, and liver injury [25]. NIK, a central regulator of the non-canonical NF-κB signaling pathway, plays a crucial role in toxin-induced liver injury. Elevated NIK expression has been reported in CCl_4_- and acetaminophen (APAP)-induced liver injury, leading to full activation of the non-canonical NF-κB signaling pathway [5,26]. In a reactive oxygen species(ROS)-dependent way, overexpression of NIK in hepatocytes exacerbates APAP-induced liver oxidative stress in mice and increases hepatocyte death and mortality. Additionally, NIK increases lipid peroxidation and cell death in primary hepatocytes treated with APAP. In contrast, hepatocyte-specific deletion of *NIK* or *IKKα* alleviates APAP-induced hepatocyte damage and promotes mice survival [26]. Interestingly, inhibition of NIK activity ameliorates toxin-induced liver injury and inflammation [6,7,72]. The specific small-molecule NIK inhibitors, B022 and XT2, ameliorate CCl_4_-induced liver inflammation, oxidative stress, and liver injury by inhibiting NIK activity, decreasing p52 protein levels and expression of cytochemokines [6,7]. Apigenin, a flavonoid found in many plants, protects against CCl_4_-induced liver injury by inhibiting the non-canonical NF-κB signaling pathway [72]. NIK acts on its function depending on its downstream signaling pathway. Recently, we have demonstrated that GBP5, a new target of NIK/p52, promotes liver injury and inflammation by inducing hepatocyte apoptosis, and deletion of *GBP5* ameliorates GalN/LPS-induced liver injury and inflammation [73].These results indicate that activation of the non-canonical NF-κB signaling pathway promotes toxin-induced chronic liver diseases, and inhibition of the non-canonical NF-κB signaling pathway, especially NIK activity, is a good approach for the treatment of toxin-induced liver injury.

### 3.5. Liver Regeneration

The liver has an enormous capacity for liver regeneration following liver injury induced by different stimuli such as drugs, viruses, alcohol, and fatty acids. It is necessary to stimulate the regenerative potential of normal hepatocytes to maintain liver physiological function [74]. However, impaired hepatocyte replication further exacerbates chronic liver diseases. Recent studies have demonstrated that the key molecules in the non-canonical NF-κB signaling pathway regulate liver regeneration through different mechanisms [27,28,29,30,31,75]. Liver injury or partial hepatectomy (PH) induces the expression of TWEAK/Fn14, BAFF, LTα/β, and LIGHT [27,28,34,76]. Deletion of *LTα*, *LTβR*, or *TWEAK*/*Fn14* in mice impairs their ability to survive PH with marked liver injury and failure to initiate DNA synthesis after PH [27,30]. Similarly, knockout of *LTβ*, *LTβR*, or *Fn14* in the mice shows decreased LPC numbers and impaired LPC-mediated liver regeneration in CDE diet-induced liver injury [28,29]. Interestingly, hepatocyte-specific deletion of *NIK* or its substrate *IKKα* significantly accelerates mouse hepatocyte proliferation and liver regeneration after PH by increasing the JAK2/STAT3 pathway [31]. These results indicate that the key molecules in the non-canonical NF-κB signaling pathway regulate liver regeneration through different mechanisms.

### 3.6. Viral Hepatitis

Viral hepatitis is a major global public health issue affecting millions of people and is linked to high morbidity and mortality [77]. Hepatitis B virus (HBV) infection induces a fast immune response in adults, leading to lifetime immunity with acute self-limited infection. However, in infants and children, life-long HBV persistence always occurs [78]. Chronic HBV and hepatitis C virus (HCV) infection both downregulate virus-specific T cell antiviral function, elevate natural killer (NK) cell cytotoxicity, and reduce antiviral cytokines production [38,79]. Key molecules of the NIK signaling pathway are increased in HBV and HCV, including NIK [32,80], LTα/β [32,33,34], LTβR [33,35], CD40L/CD40 [37], BAFF [36], OX40 [38,39], cIAP1/2 [40,41], TRAF2/TRAF3 [32,37]. During the initial state of HBV infection, *LTα*, *TRAF2*, and *NIK* have been identified among the upregulated genes, indicating that NIK activation plays a crucial role in the early HBV infection stage [32]. Serum BAFF levels are markedly increased in clinical HBV patients compared to healthy controls [36]. Activation of LTβR in HBV-infected cell lines decreases levels of HBV markers without toxicity [35]. During HCV infection *in vitro*, CD40L-CD40 signaling downregulates the effect of HCV infection and shows antiviral effects via reducing TRAF2 and TRAF3 protein levels independently of cell apoptosis [37]. HBV infection is reduced by liver-specific deletion of *cIAP1*, total deletion of *cIAP2* or antagonizing cIAP1, indicating that inhibition of cIAPs may facilitate the clearance of HBV infection [40,41]. NIK is also one of the IFN-stimulated genes [81]. NIK expression is increased in HCV-infected hepatocytes and liver tissues from chronic hepatitis C patients [42]. Overexpression of NIK promotes HCV assembly, whereas deletion of *NIK* impairs HCV particle production [42]. Therefore, NIK is essential for HCV infection, and its overexpression enhances HCV propagation. These results indicate that the key molecules in the non-canonical NF-κB signaling pathway play different roles in viral hepatitis.

### 3.7. Viral Hepatitis-Related Hepatocellular Carcinoma

HCC is the sixth most common malignancy globally and the third leading cause of human death [82]. HCC pathogenesis is associated with various etiologies, including hepatitis virus-induced HCC [83]. Emerging evidence indicates that the non-canonical NF-κB signaling pathway regulates viral hepatitis-related HCC. The key molecules such as NIK [4,47,84,85], LTα/β/LTβR [33], CD40 [43], BAFF [44], RANKL [45], OX40 [46] in the non-canonical NF-κB signaling pathway are upregulated in viral hepatitis-related HCC. Blocking CD40/CD154 signaling by the anti-CD154 antibody inhibits HCC cell proliferation [43]. Knockdown of *NIK* by specific siRNA [4] or microRNA (miR-520e) [47] reduces the level of HBV DNA, inhibits HBV-HCC cells proliferation, migration, invasion, promotes apoptosis of HBV-HCC cells, and markedly blocks the xenograft tumor growth in mice. The role of NIK in viral hepatitis-related HCC still needs further investigation.

### 3.8. Hepatic Ischemia/Reperfusion Injury

Hepatic ischemia/reperfusion disturbs liver function, leading to irreversible damage and even multiple organ failure [86]. In HIRI, CD40 [48,49,50,51,52,53], OX40 [54], RANKL [55], TRAF2 [87,88], TRAF3 [56,57,58], cIAP1 [89], RELB [59] are upregulated in the liver. CD40, as an M1 macrophage marker gene, is increased in purified Kupffer cells in mice under IR conditions [53]. The CD40L/CD40 or CD154/CD40 signaling pathway plays an important role in HIRI [50,51,52]. Deletion of *CD40L* ameliorates sinusoidal perfusion failure and reduces serum alanine transaminase (ALT) levels in the mice [48]. Neutrophils play a crucial role in the early stages of HIRI damage. Following HIRI, OX40 expression in neutrophils is increased in a time-dependent manner. In contrast, *OX40* knockout significantly ameliorates liver injury. Mechanistically, the deletion of *OX40* in neutrophils blocks the NF-κB signaling pathway through the TRAF1/2/4 and IKKα/IKKβ/IκBα pathways [54]. During HIRI, RANK protein levels are increased compared to the control group. RANK is mostly expressed in hepatocytes but not Kupffer cells [55]. Serum RANKL concentrations are steadily increased with the onset of HIRI, peaked 2 h later, and then dropped. Recombinant RANKL ameliorates HIRI [55]. TRAF3 plays an essential role in HIRI [56,57]. Hepatocyte-specific knockout of *TRAF3* ameliorates cell death and immune responses both in vitro and in vivo hepatic I/R models, whereas myeloid cell-specific deletion of *TRAF3* does not affect HIRI [58]. RELB, as another key molecule of the NIK signaling pathway, regulates HIRI progression. Silencing *RELB* by RNAi protects mice from HIRI via decreasing superoxide dismutase activity, myeloperoxidase, and TNFα production [59].These results suggest that many molecules upstream of NIK regulate HIRI progression. However, whether NIK itself regulates HIRI progression needs further study.

### 3.9. Autoimmune Hepatitis

Autoimmune hepatitis (AIH) is characterized by interface hepatitis, intrahepatic lymphocyte infiltration, high ALT and AST levels, elevated immunoglobulin G (IgG), and detection of auto-antibodies [90,91]. The NIK signaling pathway is involved in regulating AIH [60,61,92,93]. Dysregulation of T cell development isa risk factor for AIH. T cell development is controlled by thymic epithelial cells (TECs), including cortical TECs (cTECs) and medullary TECs (mTECs). The NIK/IKKα signaling pathway is highly activated in mTECs. NIK in the thymus suppresses auto-reactive CD4^+^ T cells against liver antigens [60,61]. The TEC-specific knockout of *NIK* or *IKKα* causes premature mice morbidity, reduces thymus weight and thymocyte number, induces severe liver injury, autoimmune hepatitis, and fibrosis. Mechanistically, the lack of NIK and IKKα impairs thymic medullary development and negative selection, eventually leading to the breakdown of central tolerance and generation of autoreactive T cells [60]. Whole-body, but not liver-specific or hematopoietic lineage cell-specific knockout of *NIK* causes severe liver damage, inflammation, and fibrosis. Similarly, the adoptive transfer of *NIK*-null thymus into immune-deficient mice shows similar phenotypes. Liver inflammation is caused by a large amount of CD4^+^ T cell expansion in the liver. In contrast, depletion of CD4^+^ T cells, but not CD8^+^ T cells, fully rescues liver inflammation, injury, and fibrosis in *NIK* KO mice [61]. These results suggest that thymus NIK and IKKα are required for maintaining liver function.

## 4. Future Perspectives

The role of the non-canonical NF-κB signaling pathway in liver diseases has been studied in recent years, and substantial progress has been made in the understanding of how key molecules in this signaling pathway regulate liver diseases. However, several important questions remain to be answered.

The regulation of the non-canonical NF-κB signaling pathway in liver diseases is different from immune cells in response to cytokines. The major difference is that the negative regulation of NIK by cIAP–TRAF2–TRAF3 is impaired in many liver diseases. NIK and cIAP–TRAF2–TRAF3 are both upregulated in metabolic liver diseases and viral hepatitis, which is likely due to the upregulation of their transcription. How their transcription is regulated in liver diseases needs further study. Recent studies show that m^6^A mRNA modification plays an important role in liver function, and hepatic deficiency of METTL3 (m^6^A methyltransferase) causes liver dysfunction and promotes NASH progression [94,95]. Interestingly, NIK has been shown to negatively regulate METTL3 expression [96]. Additionally, tissue-specific deletion of *METTL3* promotes inflammation [94,96,97]. It is necessary to test whether m^6^A mRNA modification regulates the expression of key molecules in the non-canonical NF-κB signaling pathway.

The degradation of NIK by cIAP–TRAF2–TRAF3 and NIK-mediated phosphorylation of IKKα and p100 are key steps in activating the non-canonical NF-κB signaling pathway. It should be noted that cIAP–TRAF2–TRAF3 are E3 ubiquitin ligases and NIK is a serine/threonine protein kinase. They may have many other targets. It is necessary to comprehensively identify their specific targets in the liver.

The liver contains multiple cell types, such as hepatocytes, cholangiocytes, hepatic stellate cells, and Kupffer cells. Liver diseases also result from the crosstalk among these cell types. It is necessary to study the contribution of the non-canonical NF-κB signaling pathway in each of these cell types.

NIK is a central and specific component of the non-canonical NF-κB signaling pathway that integrates various TNFR signals, resulting in the pathogenesis of liver injury and inflammation. Targeting NIK may be a good strategy to prevent or cure liver diseases. Two NIK inhibitors (B022 and XT2) have been shown to ameliorate liver injury and inflammation [6,7]. It is necessary to test whether they also show therapeutic effects in other liver diseases. More therapeutic small molecule inhibitors of NIK must be developed for the treatment of liver diseases.

## Figures and Tables

**Table 1:** Key molecules of non-canonical NF-κB signaling pathway regulate liver diseases.

Types of Liver Disease	Key Molecules	Dysregulation in Liver Disease	Gain-of-Function Phenotype	Loss-of-Function Phenotype	References
Hepatic glucose disorder in obesity	BAFF	Upregulated in adipocytes and in serum		Deletion of *BAFF* alters lipid distribution and insulin sensitivity, reduces adipose-tissue inflammation, fibrosis, hepatic steatosis, and lipid synthesis in hepatocytes in obesity.	[15,16]
	BAFFR			Deletion of *BAFFR* attenuates obesity and insulin resistance, reduces the number of B cells, levels of serum IgG, inflammation of visceral fat, increases expression of steatogenic genes and fatty acid deposition in the liver of obesity.	[17]
	CD40L	Upregulated in serum			[18]
	TRAF2	Upregulated in obesity	Promotes glucagon action in primary hepatocytes.	Liver-specific deletion of *TRAF2* attenuates HFD-induced hyperglycemia by decreasing HGP in response to glucagon in mice.	[1]
	TRAF3	Upregulated in the livers of obesity, hyperglycemia, and NAFLD patients	Induces hyperinsulinemia, exacerbates obesity, insulin resistance and glucose intolerance.	Liver-specific deletion of *TRAF3* decreases hyperinsulinemia, insulin resistance, glucose intolerance, and hepatic steatosis in obesity. Myeloid cell-specific deletion of *TRAF3* improves insulin resistance, glucose intolerance and hepatic steatosis and insulin signaling transduction in obesity.	[2,3,19]
	NIK	Upregulated in the livers of obesity	Promotes hepatic glucose production.	Liver-specific deletion of *NIK* improves glucose metabolism, attenuates hepatic steatosis, suppresses hepatic lipogenic program, hepatic glucose production and increases hepatic cyclic nucleotide PDE activity in obesity. Liver-specific inhibition of NIK decreases glucagon responses in obesity. Deletion of *NIK* in hepatocytes or hematopoietic lineage cells alone is insufficient to reduce hepatic steatosis in obesity.	[20,21]
	p52	Upregulated in the livers of obesity			[20]
Nonalcoholic fatty liver disease (NAFLD)	BAFF			Deletion of *BAFF* alters lipid distribution and insulin sensitivity, reduces adipose-tissue inflammation, fibrosis, hepatic steatosis, and lipid synthesis in hepatocytes in obesity.	[16]
	BAFFR			Deletion of *BAFFR* attenuates obesity and insulin resistance, reduces the number of B cells, levels of serum IgG, inflammation of visceral fat, increases expression of steatogenic genes and fatty acid deposition in the liver of obesity.	[17]
	CD40L			Deletion of *CD40L* attenuates obesity and hepatic steatosis, improves insulin sensitivity in the livers of obesity, impairs obesity-induced immune cell infiltration, glucose and lipid metabolism in adipose tissue.	[22]
	CD40			Deletion of *CD40* decreases body weight, food intake, physical activity, exacerbates diet-induced insulin resistance, increases hepatic steatosis and de novo lipogenesis in the liver, decreases liver inflammation, increases inflammation, basal lipolysis, immune cells in adipose tissue.	[23]
	TRAF3	Upregulated in the livers of obesity, hyperglycemia, and NAFLD patients	Induces hyperinsulinemia, exacerbates obesity, insulin resistance and glucose intolerance.	Liver-specific deletion of *TRAF3* decreases hyperinsulinemia, insulin resistance, glucose intolerance, and hepatic steatosis in obesity. Myeloid cell-specific deletion of *TRAF3* improves insulin resistance, glucose intolerance and hepatic steatosis and insulin signaling transduction in obesity.	[2,3,19]
	NIK			Liver-specific deletion of *NIK* attenuates hepatic steatosis, suppresses hepatic lipogenic program, hepatic glucose production and increases hepatic cyclic nucleotide PDE activity in obesity. Deletion of *NIK* in hepatocytes or hematopoietic lineage cells alone is insufficient to reduce hepatic steatosis in obesity.	[21]
	p52	Upregulated in the livers of obesity			[20]
Alcoholic fatty liver disease	NIK	Upregulated in the liver of AFLD mice and patients		Liver-specific deletion of *NIK* reduces alcoholic steatosis	[5,24]
	p52	Upregulated in the liver of AFLD mice and patients			[5,24]
Toxin-induced liver injury	LTβ	Expressed by liver progenitor cells in mice fed the CDE diet			[25]
	IKKα			Liver-specific deletion of *IKKα* ameliorates APAP-induced liver injury.	[26]
	NIK	Upregulated in the livers of APAP or CCl_4_ intoxicated mice	Aggravates APAP-induced liver injury and mortality.	Liver-specific deletion of *NIK* ameliorates APAP-induced liver injury and mortality.	[5,26]
Liver regeneration	LTα	Upregulated following PH		Deletion of *LTα* increases liver damage, reduces DNA synthesis, and mortality following PH.	[27]
	LTβ	Downregulated following PH		Deletion of *LTβ* impairs liver regeneration.	[28]
	LIGHT	Upregulated following PH	Increases hepatomegaly.		[27]
	LTβR			Deletion of *LTβR* increases liver damage, impairs liver regeneration reduces DNA synthesis, morbidity and mortality following PH.	[27,28]
	TWEAK		Increases liver progenitor cell line proliferation.	Deletion of *TWEAK* inhibits liver regeneration and progenitor cells accumulation following PH.	[29,30]
	Fn14	Upregulated following PH		Deletion of *Fn14* inhibits liver regeneration and progenitor cells accumulation following PH or under CDE feeding conditions, attenuates inflammation and liver fibrosis under CDE feeding conditions.	[29,30]
	IKKα			Liver-specific deletion of *IKKα* accelerates liver regeneration following PH.	[31]
	NIK			Liver-specific deletion of *NIK* accelerates liver regeneration following PH or in hepatotoxin-induced liver injury.	[31]
Viral hepatitis	LTα	Upregulated	Hepatocyte-specific overexpression of LTα induces chronic progressive hepatitis and hepatoxicity.		[32,33]
	LTβ	Upregulated in the livers of HBV and HCV infected patients	Hepatocyte-specific overexpression of LTα induces chronic progressive hepatitis and hepatoxicity.		[32,33,34]
	LTβR	Upregulated in the livers of HBV and HCV infected patients	LTβR activation controls HBV and decreases levels of HBV markers.	LTβR antibody reduces hepatitis development in mice.	[33,35]
	BAFF	Upregulated in HBV-infected patients			[36]
	CD40L	Upregulated in HCV infected cells	Inhibits expression of the HCV proteins, prevents replication of HCV and production of infectious viral particles, contributes to CD8^+^ T cell-mediated inhibition of viral replication in vitro.	Inhibition of CD40L partially prevents the antiviral activity of CD8^+^ T cells.	[37]
	OX40	Upregulated	Improves HBV antigen clearance and inhibits liver injury.	CD40 blockade promotes liver injury.	[38,39]
	TRAF2	Upregulated			[32]
	cIAP1/cIAP2			Liver-specific deletion of *cIAP1* or antagonizing cIAP1 or total deletion of cIAP2 controls HBV infection.	[40,41]
	NIK	Upregulated	Enhances HCV propagation, promotes lipogenesis and lipid droplet formation.	Knockdown of *NIK* decreases cytosolic lipid droplet content, and impairs HCV particle production	[32,42]
	IKKα	Upregulated		Knockdown of *IKKα* abrogates NIK-induced HCV assembly enhancement.	[42]
Viral hepatitis-related hepatocellular carcinoma	CD40/CD154			The blockade of CD40–CD154 interaction abrogates HCC cell proliferation induced by co-culturing with Bregs.	[43]
	LTα	Upregulated in human HCC			[33]
	LTβ	Upregulated in human HCC			[33]
	LTβR	Upregulated in human HCC		LTβR antibody reduces HCC development in mice.	[33]
	BAFF	Upregulated in human HCC			[44]
	RANKL	Upregulated in human HCC			[45]
	OX40	Differentially expresses in HCC, and high-OX40 expression is associated with poor survival			[46]
	NIK	Upregulated		Knockdown of *NIK* inhibits hepatoma cell growth, and reverses the enhanced proliferation mediated by anti-miR-520e.	[47]
Hepatic ischemia/reperfusion injury	CD40L			Deletion of *CD40L* ameliorates sinusoidal perfusion failure and reduces serum ALT levels in the mice.	[48]
	CD154		rmCD154 promotes hepatocytes apoptosis.	CD154 blockade inhibits immune activation, prevents T-cell infiltration, increases antiapoptotic molecules, and protects livers from HIRI.	[49,50,51,52]
	CD40	Upregulated	CD40 antibody restores HIRI.		[49,53]
	OX40	Upregulated		Deletion of *OX40* ameliorates HIRI and neutrophil infiltration.	[54]
	RANKL	Upregulated	rmRANKL attenuates HIRI, and liver injury.		[55]
	TRAF3	Upregulated	Induces cell death and inflammation.	Hepatocyte-specific deletion of *TRAF3* reduces cell death, inflammatory cell infiltration, and cytokine production.	[56,57,58]
	RELB			Knockdown of *RELB* decreases superoxide dismutase activity, myeloperoxidase, and TNFα production, and protects mice from HIRI.	[59]
Autoimmune hepatitis	NIK			Thymic-specific deletion of *NIK* induces mice die prematurely, severe autoimmune hepatitis, liver injury, and fibrosis, lung autoimmune disease, impairs B cell development and thymocyte development. Global deletion of *NIK* induces mice growth retardation, hypoglycemia, and premature death, severe liver inflammation, injury, and fibrosis, robust CD4^+^ T cell expansion in the liver.	[60,61]
	IKKα			Thymic epithelial cells-specific deletion of *NIK* induces mice die prematurely, severe autoimmune hepatitis, liver injury, and fibrosis, autoimmune lung disease, impairs B cell development.	[60]

NAFLD: non-alcoholic fatty liver disease; NASH: non-alcoholic steatohepatitis; AFLD: alcoholic fatty liver disease; NAFL: non-alcoholic fatty liver; HGP: hepatic glucose production; HBV: hepatitis B virus; HCV: hepatitis C virus; HIRI: hepatic ischemia/reperfusion injury; AIH: autoimmune hepatitis; HCC: hepatocellular carcinoma; HFD: high-fat diet; LPS: lipopolysaccharide; CCl_4_: carbon tetrachloride; NIK: NF-κB-inducing kinase; TRAF2/3: TNF-receptor-associated factor 2 and 3; cIAP1/2: cellular inhibitor of apoptosis 1/2; BAFF: B-cell-activating factor; CD40L: CD40 ligand; CD30L: CD30 ligand; LTα/β: lymphotoxin alpha/beta; OX40L: OX40 ligand; RANKL: receptor activator of NF-κB ligand; TWEAK: tumor necrosis factor-like weak inducer of apoptosis; BAFFR: B-cell-activating factor receptor; LTβR: lymphotoxin beta receptor; RANK: receptor activator of NF-κB; Fn14: FGF-inducible 14; IKKα: inhibitory kappa B kinase alpha; IgG: immunoglobulin G; PDE: phosphodiesterase; APAP: acetaminophen; PH: partial hepatectomy; CDE: choline-deficient, ethionine-supplemented; Breg: regulatory B cell; ALT: alanine transaminase; ROS: reactive oxygen species.

## Data Availability

Not applicable.
